# Fatigue after transient ischemic attack: cross-sectional associations with comorbidities, activities of daily living and social participation

**DOI:** 10.3389/fneur.2026.1870184

**Published:** 2026-10-26

**Authors:** Gustaf Magaard, Fredrik Öhberg, Per Liv, Ann Sörlin, Britt-Marie Stålnacke, Xiaolei Hu

**Affiliations:** 1 Department of Community Medicine and Rehabilitation, Umeå University, Umeå, Sweden; 2 Department of Diagnostics and Intervention, Biomedical Engineering and Radiation Physics, Umeå University, Umeå, Sweden; 3 Department of Public Health and Clinical Medicine, Umeå University, Umeå, Sweden

**Keywords:** activities of daily living, cross-sectional study, depression symptom, fatigue, long-term outcome, sleep problem, social participation, transient ischemic attack

## Abstract

**Background and objectives:**

Fatigue is a common symptom during the first year after transient ischemic attack (TIA), but its long-term persistence remain unclear. This study aim to assessed long-term perceived fatigue after TIA and to evaluated associated comorbidities and functional consequences.

**Materials and methods:**

This observational and cross-sectional study included 431 community-dwelling adults assessed 1–13 years after TIA in an outpatient setting, with a 25% response rate. Fatigue was measured using the Fatigue Assessment Scale in three subgroups: first-ever TIA (*n* = 299), recurrent TIA (*n* = 54), and TIA with stroke (*n* = 78). Depression and anxiety were evaluated using the Hospital Anxiety and Depression Scale, and. An activities of daily living and social participation using were determined using the Stroke Impact Scale. Multivariable ordinal linear regression analyses were performed in the first-ever TIA group to examine associations between fatigue, comorbidities and functional outcomes.

**Results:**

In the first-ever TIA group, fatigue levels were similar across time-since-TIA strata up to 13 years (mean 21.8, standard deviation [SD] 5.2); 35% exceeded the fatigue cut-off. Fatigue in the first-ever TIA group was lower than in the recurrent TIA group (mean 23.9, SD 5.7; *p* < 0.05) and TIA with stroke group (mean 23.8, SD 6.5; *p* < 0.05). Amon participants with first-ever TIA, depression (β = 6.8, 95% confidence interval [CI] 3.5–10.1; *p* < 0.001), severe sleep problem (category 4: β = 3.9, 95% CI 0.9–6.8; *p* = 0.010) and (category 5: β = 5.9, 95% CI 2.2–9.6; *p* = 0.002), atrial fibrillation (β = 2.1, 95% CI 0.3–3.9; *p* = 0.02) and heart disease (β = 1.8, 95% CI 0.2–3.4; *p* = 0.03) were associated with higher fatigue. Higher fatigue was also associated with reduced activities of daily living (β = 0.15, 95% CI 0.07–0.22, *p* < 0.001) and lower social participation (β = 0.06, 95% CI 0.02–0.10; *p* = 0.004).

**Conclusion:**

Fatigue affected more than one-third of first-ever TIA survivors and did not differ materially across cross-sectional time-since-TIA strata up to 13 years. Greater fatigue was associated with depressive symptoms, severe sleep problems, atrial fibrillation, heart disease, and reduced daily activity and social participation. Early identification and management of fatigue and related comorbidities may improve functioning. Longitudinal studies are needed to determine temporal relationships and whether targeted management improves outcomes.

## 1 Introduction

A transient ischemic attack (TIA) has historically been defined as a vascular event in the central nervous system with focal neurological symptoms based on the symptom-duration lasting < 24 h (1). TIA incidence is 1.19 per 1,000 person-years in Western countries (2). Individuals with a first-ever TIA have an approximately 5-fold higher risk for ischemic stroke onset than those without TIA (2). In Sweden, approximately 9,000 people experienced a TIA in 2023 (3) based on the historical TIA definition due to limited resource on using magnetic resonance imaging for TIA diagnose, even TIA has been redefine using tissue-based approach in 2009 (4). However, clinical diagnosis, risk assessment and secondary preventions for person with TIA in the acute phase have been well established in Sweden (5, 6).

Several TIA sequelae, such as fatigue and psychological and cognitive impairments, have been reported with increased interest over the last 15 years (7–9). Among the various TIA sequelae, fatigue has attracted particular interest in recent years (10–13). According to large cohort studies, patients with a TIA have an increased risk of 43% for experiencing fatigue within 19 months compared to the age-matched controls (14); and pathologic fatigue is common up to 12 months after TIA if patients report fatigue within 14 days after discharge (15). However, fatigue beyond 12 months after a TIA has been insufficiently studied.

Despite extensive studies on post-stroke fatigue (16–19) including assessments using the Fatigue Assessment Scale (FAS) (20), few studies have examined fatigue after TIA using the FAS. FAS is a short and established patient-reported measure of perceived fatigue in neurological populations, including individuals after stroke. Compared with other commonly used tools, such as fatigue severity scale and Fatigue impact scale, FAS measures primarily general perceived fatigue than a multidimensional fatigue or focusing impact of fatigue (19, 20). Post-stroke fatigue could be multifactorial because many comorbidities, such as diabetes, hypertension, atrial fibrillation, and heart disease, may contribute to fatigue (21). Our previous study demonstrated that broadly perceived disabilities play a significant role in everyday life and recovery among community-dwelling individuals up to 13 years after a TIA (22). However, the potential cross-sectional associations between fatigue and various comorbidities as well as functioning among individuals after a TIA have not been fully elucidated.

Therefore, the primary aim of this study was to investigate the level of perceived fatigue among patients after a TIA at different time intervals after TIA onset, ranging from several months up to 13 years and identify subgroup differences. A secondary aim was to investigate cross-sectional associations between fatigue and comorbidities and demographic factors. We additionally explored associations between fatigue and activities of daily living (ADL) and social participation.

## 2 Materials and methods

### 2.1 Study design and patients

This observational and cross-sectional study included community-dwelling adults aged ≥18 years admitted to the Stroke Center at the University Hospital of Umeå with a TIA diagnosis between January 2005 and June 2018. Physicians determined all TIA diagnoses according to the symptom-based diagnosis of TIA: “rapidly developed clinical signs of focal or global disturbance of cerebral function, lasting less than 24 hours, with no apparent non-vascular cause” (23).

Medical records were screened to exclude patients with other central nervous system (CNS) diagnoses, including brain tumors, epilepsy, sinus thrombus, as well as those who had died or were unable or unwilling to provide written informed consent or complete the questionnaires.

This cross-sectional study partially overlaps with a previous publication that reported detailed Stroke Impact Scale data among participants with first-ever TIA participants (*n* = 299) (22). In the current study, we focused on the fatigue and include not only participants (*n* = 299) with first-ever TIA but also those with recurrent TIA and those with a history of both TIA and stroke. In addition, we examine the associations between fatigue and comorbidities, ADL and social participation, extending the analyses reported in the previous study (22).

### 2.2 Ethics

This study was approved by the Regional Ethical Review Board in Umea, Sweden (Dnr 2016-355-32 M). Written informed consent was obtained from all the participants. This study conformed to the principles of the Declaration of Helsinki.

### 2.3 Data collection and questionnaires

Each participant was sent a letter containing the study information, written informed consent form, demographic data collection form, and the questionnaires described in this section with a return envelope (sent in August 2018). If a returned questionnaire contained considerable missing data, such as missing answer in one of the questionnaires, same questionnaire was resent to the participant to obtain the missing information. If the returned questionnaire contained minimal missing data, such as missing answer in one or few questions within a questionnaire, a research team member contacted the participant by telephone to obtain the missing information.

Information on comorbidities and demographic characteristics, were obtained from patient medical records. They were included heart disease, hypertension, diabetes mellitus, hyperlipidemia and atrial fibrillation. Each condition was coded as a binary variable (present/absent) based on documented diagnosis in the medical records. Body mass index (BMI) was calculated (kg/m^2^) using participant self-reported data and categorized as normal weight (18.5–25), overweight (>25), or underweight (<18.5) (24).

The Fatigue Assessment Scale (FAS) is a 10-item questionnaire with five questions on physical fatigue and five on mental fatigue (25) that are answered using a five-item Likert scale, ranging from “Never” to “Always.” The developers mixed the physical and mental fatigue questions, and two questions are reverse scored to maintain their validity. The total FAS score ranges from 10 to 50. It has been used and validated for many diseases, including stroke and TIA (11, 26), and is considered unidimensional. The FAS has demonstrated high and good internal consistency and convergent validity in a Swedish version (27) without floor or ceiling effects (28) or sex bias (25). Two FAS score cutoffs have previously been reported (22 and 24 points) to define fatigue (28); we chose 24 points for this study even we used the continuous FAS score as the primary outcome and the proportion above the prespecified cut-off 24 as a secondary descriptive results in the figure. The result while the proportions above cut-off 22 were also presented in the result. FAS scales were analyzed as the continuous variables.

To decrease participants’ burden on answering too many question, sleep problems were evaluated using three brief, self-report questions from our previous study (29, 30): Question 1: problems to fall asleep; Question 2: Problems to maintain sleep; and Question 3: Sleeping makes for needed rest. These questions were clinically informed and routinely explored during patient-physician consultations. They were intended to capture common and clinically relevant dimensions of sleep problem, rather than provide a comprehensive assessment of sleep disorders. The questions were answered on the same Likert scale as the FAS. Each question is to be answered on a scale from 1 (all of the time) to 5 (none of the time). Subsequently, the score was converted to a value between 0 and 100 using a linear transformation, where 0 was the worst condition and 100 was the best (same transformation as used for the Stroke Impact Scale [SIS]). The sleep scores were analyzed as the category variables.

The Hospital Anxiety and Depression Scale (HADS) is a 14-item questionnaire that takes 2–5 min to complete and records depression and anxiety during “the past week.” Answers are provided using a 4-point Likert scale. Scores range from 0 to 21 for depression and 0–21 for anxiety (31). Scores of 8–10 suggest the presence of the state and higher values indicate probable presence. HADS subscales were analyzed as the continuous variables.

The Modified Rankin Scale questionnaire (mRSq) is a standardized, pragmatic, validated tool to help reliably score a person’s disability (32). Five questions are answered with “yes” or “no” by the individual. A scale rating from 0 (no symptoms) to 5 (total physical dependence) is rated using mRSq flowchart; mRSq scores ≤2 are considered to represent functional independence. The mRSq were analyzed as the category variables.

The SIS comprises 59 questions representing eight domains (33): strength, memory and thinking, emotions, communication, mobility, hand function, ADL/instrumental ADL (IADL), and social participation. Each question is answered using a Likert scale ranging from 1 (significant impairment) to 5 (no impairment). The initial score is converted to score between 0 and 100 for each domain, 100 represents the best condition (no problems) and 0 represents the worst condition. The SIS results in this cohort have been previously reported (22). This study only used the ADL/IADL and social participation domains in a multiple linear regression analysis.

### 2.4 Data management, presentation, and statistical analyses

All collected data were pseudonymized before processing (all names, personal identification numbers, and addresses were deleted). Each participant was then assigned a unique identification. The continuous variables for the demographic data are presented as the mean ± standard deviation. The categorical variables such as sex and comorbidities are presented as frequencies [number of the subgroup/ number of total cohort (n/N) and percentages].

All collected data were stored in compliance with the regulations of Umeå University. If any data were missing, the corresponding case was excluded from statistical analyses. The participants were stratified into three subgroups: first-ever TIA as the index event (patients with only one TIA event), recurrent TIA (multiple TIA events), and history of both TIA and stroke (stroke before or after TIA) in order to compare the fatigue severity in these groups. To explore potential patterns related to times elapsed since TIA onset, participants within the first-ever TIA group was further stratified into three subgroups based on time-since-onset: <1 year, 1–5 (<5) years, and 5–13 years since a first-ever TIA.

Kruskal–Wallis tests were used to compare fatigue in three different TIA subgroups. The same procedure was carried out between three time-periods, respectively. Mann–Whitney U-tests were used for the between group comparisons. Each *p* value was adjusted to account for multiple comparisons using Bonferroni correction.

Multivariable linear regression analyses were used to examine the associations between fatigue and the comorbidities and demongraphic factors, as well as the associations between fatigue and ADL and participation. FAS was specified as the dependent variable, and each independent factor were evaluated in separate models. The results were reported as β coefficients, 95% confidence intervals (CIs), and *p*-values.

For the multivariable analyses, covariates were selected priori based on directed acyclic graphs (DAG) constructed for each comorbidity (Table 1). DAG provide a visual and conceptual framework for specifying assumptions about the relationships among exposure, outcomes and covariables (34). They were used to identify appropriate adjustment sets based on the assumed causal structure, while avoiding adjustment for potential mediators or colliders. DAGs was used to inform adjustment for potential confounding and to improve the transparency and reproducibility of covariate selection (35). Constructed DAGs are presented as Supplementary Figure S1.

**Table 1:** Presentation of the adjustment variables for each comorbidities or factors.

Variables	Age	Sex	BMI	Anxiety	Depression	Sleep problems	Heart disease	Hyper-tension	Hyper-lipidemia	Diabetes	Atrial fibrillation
Anxiety		X		—	X	X	X				
Depression				X	—	X					
Sleep problems	X			X	X	—	X	X		X	
Heart disease	X		X	X	X		—	X			
Hypertension	X	X	X	X	X		X	—			
Hyperlipidemia	X		X					X	—	X	
Diabetes	X	X	X	X	X		X	X		—	
Atrial fibrillation	X							X			—
Sex	X	—	X	X	X	X	X	X			

X: indicated adjusted use of variables (top row) in statistic calculation. – indicated not applicable in calculation.

Normality assumption of residuals from linear regression models was assessed using histograms and Q-Q plots, and no major violations were observed. Statistical analyses were performed using the IBM SPSS Statistics software (version 27; IBM Corp., Armonk, NY, USA). The figures were created using GraphPad Prism software (version 9; San Diego, CA, USA). Significance level was set at *p* < 0.05.

## 3 Results

### 3.1 Recruitment of participants and response rate

A total of 1,894 adult patients diagnosed with a TIA at the Stroke Center of the University Hospital of Umeå were comprehensively assessed to ascertain their suitability for this study (Figure 1). Of these, 481 provided written informed consent to participate in the study (response rate 25%). After excluding those with other CNS related diagnoses, a total of 431 persons met the study eligibility criteria, including individuals with a first-ever TIA (*n* = 299), recurrent TIA (*n* = 54), and a TIA with stroke (*n* = 78). In the first-ever TIA group, most participants (93%) had a TIA more than 1 year prior.

**Figure 1:**
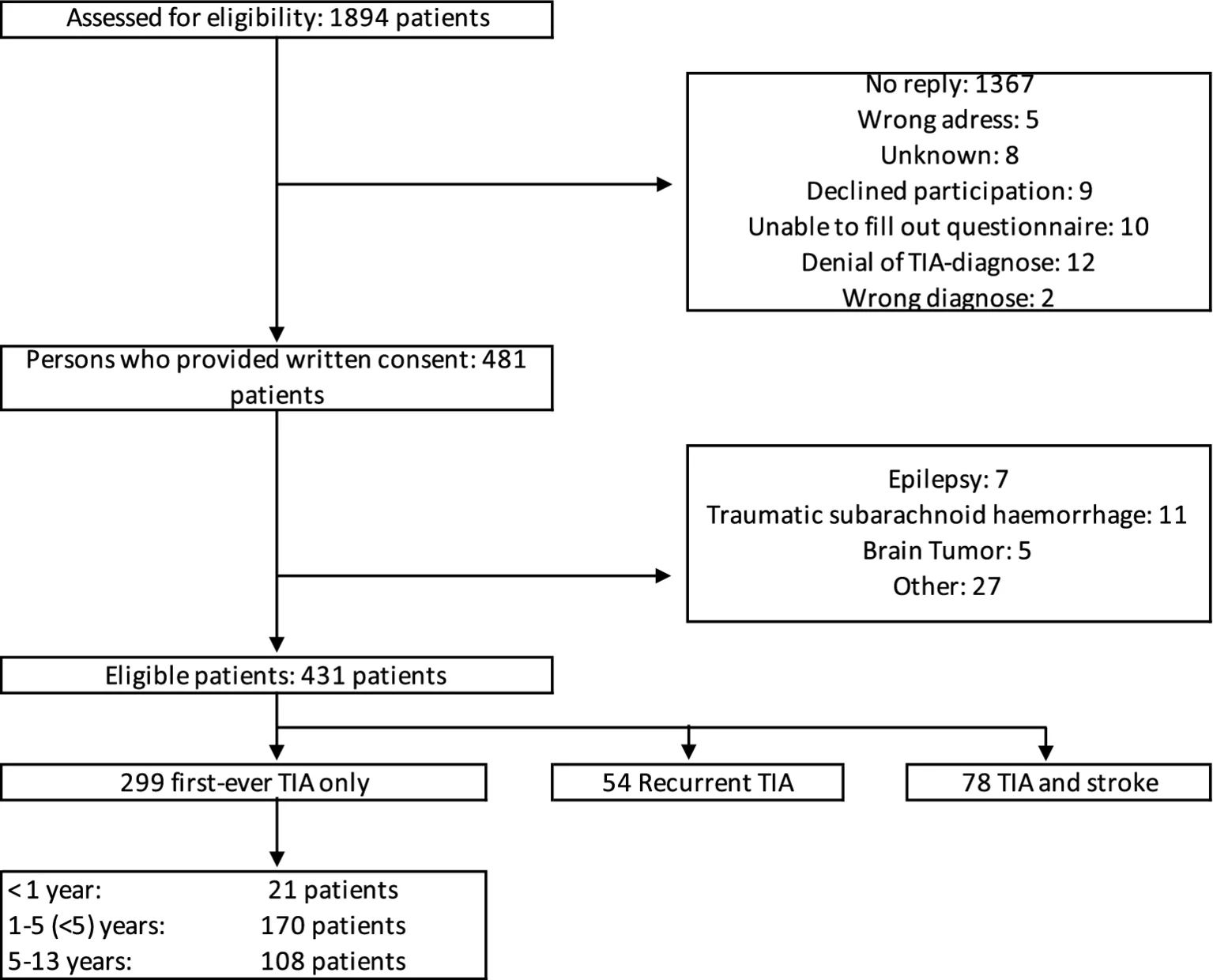
Flowchart of the inclusion process and numbers of the participants in the subgroups. “TIA and Stroke” indicates the history of both TIA and stroke group.

### 3.2 Comorbidities and demographic characteristics

The participant demographic characteristics are summarized in Table 2. Most participants (93%) had a TIA more than 1 year before the data were collected. The mean age of the patients was 73 ± 11 years. Most were males (62%), with slightly over one-third females. The mean age was 4 years’ higher in the TIA with stroke group than in the first-ever TIA group. The recurrent TIA and TIA with stroke groups comprised fewer females compared to the first-ever TIA group. BMI scores were higher in the first-ever TIA group (58% considered overweight) and recurrent TIA group (62% considered overweight) than in the TIA with stroke group (49% considered overweight). The demographic data for the patients with first-ever TIA have been reported in our previous study (22).

**Table 2:** Demographic and co-morbidity data of the cohort.

Patient characteristics	Total (*n* = 431)	First-ever TIA (*n* = 299)	Recurrent TIA (*n* = 54)	TIA and stroke (*n* = 78)
Year after transient ischaemic attack, mean (SD)	4.8 (3.6)	4.8 (3.7)	4.7 (3.3)	5.0 (3.4)
Age, years, mean (SD)	73 (11)	72 (11)	73 (9)	76 (9)
Sex, male/female, *n*	266/165	168/131	40/14	58/20
BMI, mean (SD)	25.6 (7.4)	26.3 (4.0)	26.3 (3.8)	26.6 (4.0)
Comorbidities, cases, *n/N* (%)				
Hypertension, *n*/*N* (%)	248/431 (58)	164/299 (55)	34/54 (63)	50/78 (64)
Diabetes, *n*/*N* (%)	56/431(13)	36/299 (12)	7/54 (13)	13/78 (17)
Atrial fibrillation, *n*/*N* (%)	52/431 (12)	34/299 (11)	4/54 (7)	14/78 (18)
Hyperlipidaemia, *n*/*N* (%)	124/431 (29)	75/299 (25)	14/54 (26)	35/78 (45)
Heart diseases, *n*/*N* (%)	71/431 (17)	42/299 (14)	11/54 (20)	18/78 (23)
Modified Rankin Scale questionnaire, mean (SD)	0.5 (1.0)	0.4 (0.9)	0.4 (0.7)	1.1 (1.4)
HADS depression, mean (SD)	2.5 (2.8)	2.1 (2.3)	3.5 (4.0)	3.3 (3.4)
HADS anxiety, mean (SD)	2.9 (3.0)	2.8 (2.9)	3.6 (3.1)	3.0 (3.5)
Sleep problems, mean (SD)	61 (18)	62 (18)	59 (15)	61 (22)

n/N, number of case/Number of the cohort; SD, standard deviation.

Diabetes was observed in 13% of the whole cohort, with 17% in the TIA with stroke group. Hypertension was the most common comorbidity with 58% in the study cohort being affected with 63% in the recurrent TIA group and 64% in the TIA with stroke group, respectively. Atrial fibrillation was more common in the TIA with stroke group (18%) than in the first-ever TIA (11%) and recurrent TIA (7%) groups. Heart disease was observed mostly in the TIA with stroke group (20%) and to a lesser extent in recurrent TIA and first-ever TIA groups. Hyperlipidemia was commonly observed in the study population (approximately one-third of the study population); in the TIA with stroke group close to half were affected (45%); and in the other two groups results were closer to one-fourth of the population.

Most patients in the first-ever TIA group (93%) and the recurrent TIA group (98%) were considered to have total independence (mRSq scores ≤2). The TIA with stroke group had less independence (81%). The HADS mean scores for depression and anxiety were below the cutoff (<8 points) in all three groups; few persons with depression/ anxiety symptoms (above the cutoff) were in the first-ever-TIA group (4%/ 6%), recurrent TIA group (13%/15%), and the TIA with stroke group (13%/13%). Affected sleep was common in all three groups.

### 3.3 Perceived fatigue

The mean FAS score for all participants was 22.4 ± 5.6. The FAS scores of the first-ever TIA group (21.8 ± 5.2) were lower those of the cut-off (≥24) for fatigue, although 35% of this group has scores above the prespecified cut-off. Half of the recurrent TIA group and 40% of the TIA with stroke group had scores greater than the cutoff for fatigue. The latter groups demonstrated significantly higher perceived fatigue than the first-ever TIA group (Figure 2A).

**Figure 2:**
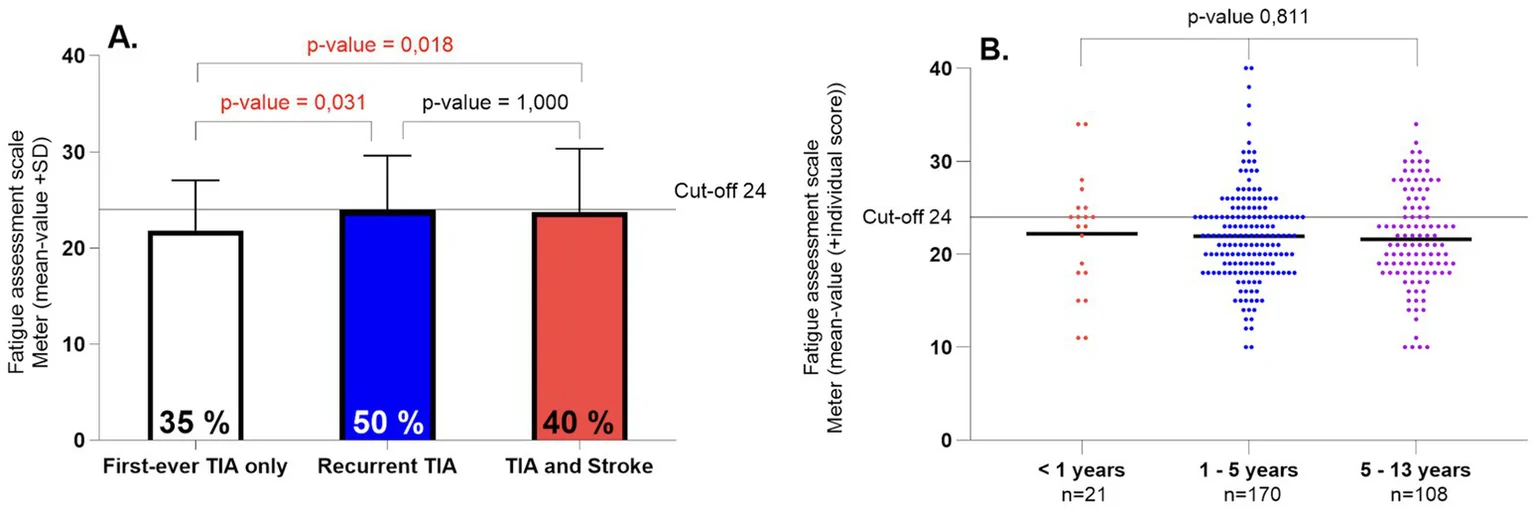
Perceived fatigue after TIA. **(A)** The bar graph presents perceived fatigue assessed by the FAS for the three subgroups with the percentages of fatigued individuals in each subgroup. **(B)** The plot graph shows perceived fatigue in mean scores (lines) and individual scores (dots) for the first-ever TIA group stratified over 13 years after TIA. FAS, Fatigue Assessment Scale; TIA, transient ischemic attack. “TIA and Stroke” indicates the history of both TIA and stroke group.

In addition, when applying the cut-off (≥22), more participants were classified with fatigue in first-ever TIA group (51%), recurrent TIA group (67%) and history of both TIA and stroke group (60%).

A similar level of perceived fatigue was demonstrated among patients after a first-ever TIA stratified across time-since-onset, < 1 year (22.1 ± 5.2), 1 to 5 years (22.0 ± 5.3) and 5–13 years (21.4 ± 5.0) (Figure 2B).

### 3.4 Associations between comorbidities and perceived fatigue

Among participants in the first-ever TIA group, higher perceived fatigue was significantly associated with atrial fibrillation, and heart disease severe sleep problems and depressive symptoms in the multivariable linear regression model adjusted for age (Table 3). Compared with participants reporting the best sleep (category 1), those reporting severe sleep problems had higher fatigue scores in both sleep category 4 (β = 3.9) and sleep category 5, representing the worst sleep (β = 5.9). Higher levels of depressive symptoms were associated with substantially higher fatigue scores (β = 6.8). Atrial fibrillation (β = 2.1) and heart disease (β = 1.8) were also associated with higher fatigue scores. There were no significant associations between perceived fatigue and sex, diabetes, hyperlipidemia, hypertension or anxiety (Table 3).

**Table 3:** Cross-sectional associations between comorbidities and factors, and perceived fatigue among the patients with a first-ever TIA.

Variable	*β*	95% CI	*p*
Female sex^1^	0.589	[−0.557, 1.735]	0.313
Hypertension^1^	0.134	[−1.092, 1.361]	0.829
**Atrial fibrillation**^1^	**2.089**	**[0.293, 3.886]**	**0.023**
Hyperlipidemia^1^	−0.51	[−1.862, 0.843]	0.459
Diabetes^1^	1.163	[−0.585, 2.910]	0.191
**Heart disease**^1^	**1.778**	**[0.160, 3.396]**	**0.031**
Sleep_category = 1 (best) ^1^	reference		
Sleep_ category =2^1^	0.845	[−1.002, 2.693]	0.369
Sleep_ category =3^1^	1.582	[−0.275, 3.439]	0.095
**Sleep_ category =4**^1^	**3.883**	**[0.933, 6.834]**	**0.01**
**Sleep_ category = 5**^**1**^ **(worst)**	**5.922**	**[2.195, 9.648]**	**0.002**
**HADs depression**^2^	**6.807**	**[3.470, 10.144]**	**<0.001**
HADs anxiety^2^	1.765	[−0.863, 4.393]	0.187

Bold text indicates the significant association. β regression coefficient, 95% CI, 95% confidence intervals, *p*, *p* values, were analyzed using multivariate linear regression after adjustment for age.

1 β represents a slope.

2 β presents group difference.

### 3.5 Associations between perceived fatigue and ADL and social participation

After adjustment for age, higher patient-reported fatigue was significantly associated with lower ADL/IADL scores (β = 0.15) and lower social participation scores on the SIS (β = 0.06) in multivariable linear regression analyses (Table 4).

**Table 4:** Cross-sectional associations between perceived fatigue and daily activities and social participation among the patients with a first-ever TIA.

Variable	β	95% CI	*p*
ADL	**0.146**	**[0.074–0.219]**	**<0.001**
Social participation	**0.059**	**[0.019–0.099]**	**0.004**

Bold text indicates the significant association. β regression coefficient representing a slope, 95% CI, 95% confidence intervals, *p*, *p* values, were analyzed using multivariate linear regression after adjustment for age.

## 4 Discussion

This observational and cross-sectional study examined perceived fatigue among 431 community-dwelling participants after a TIA using the FAS. We found that 35% of the participants in the first-ever TIA group experienced perceived fatigue according to the selected cut-off (≥ 24), even though their fatigue scores mean in the whole cohort was just below the cutoff for fatigue. Perceived fatigue did not differ materially across cross-sectional time strata, that is several months up to 13 years post-TIA. Patient-reported fatigue was significantly higher among those who experienced recurrent TIA or TIA with stroke than among those who experienced their first-ever TIA. More perceived fatigue was associated with greater depression, severe sleep problems, heart disease, and atrial fibrillation. Notably, higher patient-reported fatigue was significantly associated with lower daily activities and SIS social participation scores among persons with first-ever TIA.

Approximately one-third of the participants who experienced their first-ever TIA event reported fatigue above the selected cut-off. This finding is consistent with that of our pilot study (36) and another study assessed fatigue by FAS in a cohort with TIA and minor stroke (9). However, the proportion of the fatigue above the prespecified cut-off in the present study is relatively lower compared to a recent prospective study reported 40%–50% of participants with fatigue above the prespecified cut-off within first year after TIA (9, 15). These discrepancies may partly cause by different assessments used in the studies and a higher cut-off chosen in the current study. Notably, participants in the TIA with stroke group had significantly higher FAS scores than those in the first-ever TIA group. This is consistent with other studies that reported relatively higher FAS scores among patients who experienced a minor stroke in the acute to subacute stages after stroke (11). These data indicate that perceived fatigue levels may partially correlate with the relative older age, higher stroke-related disability and comorbidity burdens as well as sleep disturbances and depression in the TIA and stroke group.

It was somewhat unexpected that perceived fatigue levels were similar from several months up to 13 years post-TIA among our community-dwelling participants. Theoretically, even if a TIA causes ischemic damage, the minor damaged brain can be repaired through neuroplasticity over time (37). Therefore, fatigue is expected to be alleviated in the subacute and chronic phases after TIA. Meanwhile, we found that the perceived fatigue levels over time were similar to those reported in patients with stroke (16), despite the severity of fatigue being less extensive after a TIA. Although we could not rule out minor strokes (4–6, 38) in this cohort since a clinical, rather than radiological-based, TIA diagnosis was used. This persistent post-TIA fatigue may reflect underlying multifactorial mechanisms similar to those observed in post-stroke fatigue (12, 19).

We further investigated whether various factors including basic demographics and comorbidities played any role in long-term patient-reported fatigue after TIA by multivariate linear regression analyses. We found that greater depression, but not anxiety, was independently associated with higher perceived fatigue scores after a first-ever TIA. This result is in line with the finding of a significant negative association of depression with post-stroke fatigue (18). The present data indicated strongly an importance of identifying and treating depression among persons with TIA. Markedly, severe sleep problems demonstrated significant association with more fatigue. This is obviously logical and consistent with the finding in post-stroke fatigue (39). Unlike post-stroke fatigue (21), hypertension was not associated with post-TIA fatigue in the present study. Furthermore, heart disease and atrial fibrillation had an increased β of fatigue in this study and similar results have been demonstrated for heart disease (40). Our data indicated a close relationship between post-TIA and post-stroke fatigue, and possibly an overlap with fatigue among persons with coronary heart disease. Together, the current results thus suggested that post-TIA fatigue appears to be associated with multiple comorbidities beyond the TIA event alone.

Notably, elevated perceived fatigue levels had a significant adverse association with ADL and social participation among the participants after a first-ever TIA. The present data are consistent with another Swedish study (12) and our previous study from a similar cohort where patient-reported disabilities, including emotion and strength, showed low to moderate associations with ADL and social participation (22). Together with similar findings among persons with stroke, our data imply that a multi-professional follow-up to manage the treatable comorbidities may be useful in the clinical praxis to reduce their adverse effects on daily activities and social participation and to improve overall recovery after a TIA. However, such clinical rehabilitation interventions for individuals with TIA are currently lacking in the routine clinical practice in Sweden (6).

This study has several strength. First, it included a relatively large, community-based sample of 431 participants, which is substantial compared with many previous studies and provided sufficient statistical power for the multivariable regression analyses. Second, participants were recruited across a broad time frame after the TIA onset, allowing assessment of fatigue across over a decade, although the cross-sectional design does not permit conclusion about changes in fatigue over time. Third, the study used established patient-reported outcome measures on various functioning and clinically documented comorbidities strengthen the characterization of participants and adjustment for potential confounding.

Meanwhile, several limitations in this study should be considered. The response rate was relatively low (25%), which raises the possibility of selection- and healthy-survivor biases. Baseline comparisons between responders and non-responders could not be performed due to the ethical and data-protection reasons for the non-responders. Individuals with more severe fatigue, cognitive impairment, poorer health, or greater disability may have been less likely to complete the questionnaires, which potentially resulted in an underestimation of post-TIA fatigue and its associations. Conversely, patients with a high interest in their symptoms may have been more motivated to respond.

The cross-sectional design is another important limitation. Although DAG were used to guide covariate selection based on prespecified assumptions about potential confounding (34), this study design limits ability to establish temporal alteration of fatigue and relationships between fatigue and some covariates, particularly depression symptom and sleep problems. Therefore, the observed associations should not be interpreted as causal, and reverse causal and residual confounding cannot be excluded. Meanwhile, there was no pre-TIA assessment to fatigue and no health control group, which makes it impossible to the causal relationship between TIA and fatigue or differed from fatigue from the general population. Furthermore, the diagnosis of TIA was based on clinical symptom assessment, and some participants with minor stroke may therefore have been misclassified as having TIA. Although the FAS has been tested, validated, and used in the stroke field (20, 27, 28), it remains a subjective measure with general limitations (25, 26). More specific and objective measurements are required to improve fatigue identification (41) in the future. In addition, the sleep composite measure was not fully validated, which may have introduced measurement error.

Finally, the present study partly overlaps with a previous reported cohort (22). Although the current analysis addresses a different research question and focuses specifically on fatigue and its associations with comorbidities and functional outcomes, this overlap should be considered when interpreting the findings and their contribution to the existing literature.

In conclusion, fatigue affected more than one-third of first-ever TIA survivors and did not differ materially across cross-sectional time-since-TIA strata up to 13 years. The greater fatigue was associated with elevated depressive symptoms, severe sleep problems, atrial fibrillation, heart disease, and reduced daily activity and social participation. Our results suggested that early identification and management of fatigue and related comorbidities the clinical praxis may improve functioning among individuals with TIA. Longitudinal studies are needed to determine temporal relationships and whether targeted management improves outcomes.

## Data Availability

The datasets presented in this article are not readily available because the raw data supporting the conclusions of this article will be made available by the correspondent author, upon reasonable request. Requests to access the datasets should be directed to xiaolei.hu@umu.se.

## Supplementary material

SUPPLEMENTARY FIGURE S1 Directed acyclic graph (DAG) analyses. Outcome marked with blue colored black circle, exposure marked with green colored black circle. Blue colored circles indicated other potential confounders and mediators. White colored circles indicate adjusted variables while red colored circles indicate no need for adjustment. All DAGs were named by the exposure.
